# A train-the-trainer education and promotion program: chronic fatigue syndrome – a diagnostic and management challenge

**DOI:** 10.1186/1472-6920-8-49

**Published:** 2008-10-15

**Authors:** Dana J Brimmer, K Kimberly McCleary, Teresa A Lupton, Katherine M Faryna, Kevin Hynes, William C Reeves

**Affiliations:** 1 Division of Viral and Rickettsial Diseases, Centers for Disease Control and Prevention, Atlanta, GA, USA; 2 CFIDS Association of America, Charlotte, NC, USA; 3 Illinois AHEC Program, Midwestern University, Downers Grove, IL, USA

## Abstract

**Background:**

Chronic fatigue syndrome (CFS) is a complicated illness for providers and patients. Fewer than 20% of persons with CFS have been diagnosed and treated. For providers, compounding the issue are the challenges in making a diagnosis due to the lack of a biomedical marker.

**Methods:**

The objective of the CFS diagnosis and management curriculum was to instruct core trainers as to the evaluation, diagnosis, and management of CFS. Over a two year period, 79 primary care physicians, physician assistants, and nurse practitioners from diverse regions in the U.S. participated as core trainers in a two day Train-the-Trainer (TTT) workshop. As core trainers, the workshop participants were expected to show increases in knowledge, self-efficacy, and management skills with the primary goal of conducting secondary presentations.

**Results:**

The optimal goal for each core trainer to present secondary training to 50 persons in the health care field was not reached. However, the combined core trainer group successfully reached 2064 primary care providers. Eighty-two percent of core trainers responded "Very good" or "Excellent" in a post-tessurvey of self-efficacy expectation and CFS diagnosis. Data from the Chicago workshops showed significant improvement on the Primary Care Opinion Survey (p < 0.01) and on the Relevance and Responsibility Factors of the CAT survey (p = 0.03 and p = 0.04, respectively). Dallas workshop data show a significant change from pre- to post-test scores on the CFS Knowledge test (p = 0.001). Qualitative and process evaluation data revealed that target audience and administrative barriers impacted secondary training feasibility.

**Conclusion:**

Data show the workshop was successful in meeting the objectives of increasing CFS knowledge and raising perceived self-efficacy towards making a diagnosis. The CFS TTT program informed an educational provider project by shifting the format for physicians to grand rounds and continuing medical education design while retaining TTT aspects for nurse practitioners and physicians assistants. Evaluations also indicate that secondary trainings may be more readily employed and accepted if administrative barriers are addressed early in the planning phases.

## Background

Chronic fatigue syndrome (CFS) is a complex illness characterized by medically and psychiatrically unexplained disabling fatigue that is not relieved by rest and is accompanied by symptoms of prolonged post-exertion malaise, unrefreshing sleep, impaired concentration or short-term memory, muscle or joint pain, headache, sore throat and tender lymph nodes [1]. CFS affects at least 4 million adults in the United States [2-4]. Most people with CFS have been ill between 5 and 7 years and at least a quarter of them are unemployed or receiving disability because of the illness [4-7]. The average affected family forgoes $20,000 annually in lost earnings and wages (approximately half of the median United States household income) [6]. Despite the protracted chronic nature of CFS and severity of the associated impairment and disability, fewer than 20% of persons with CFS have been diagnosed and treated by a physician [3,4].

CFS presents a unique diagnostic and management challenge to health care providers. The etiology, pathophysiology and risk factors for CFS remain inchoate; there are no pathognomonic physical signs or diagnostic laboratory abnormalities; and treatment is targeted at ameliorating symptoms rather than definitive cure [7]. Difficulties with diagnosis and medical management are compounded because, in spite of functional impairment, patients often do not appear to be ill; suffer comorbid emotional distress; and frustrated by lack of diagnosis; go from physician to physician [8]. Lack of timely CFS diagnosis delays intervention, which results in increased morbidity. Education of primary health care professionals about CFS is critical to the effective detection, diagnosis, and care of persons with the illness.

The education of health care providers poses a challenge that has long been recognized. Following graduation, practicing health care providers pursue continuing medical education (CME) as a means of maintaining competency, keeping abreast of new technology, and meeting requirements for re-licensure [9]. Traditional examples of CME include conferences, workshops, and grand rounds. No single method is clearly more effective to enhance practitioners' performance [10-12]. The traditional forums provide an opportunity to learn new skills and incorporate new perspectives on disease management in clinical practice [13] but they generally present didactic material and do not include an interactive component, which has been shown to change behavior [10].

One example of provider education that incorporates interactive methods is the train-the-trainer (TTT) model. In this model, experienced health care providers use educational materials and information to teach other providers (the core trainers), who then return to their workplace or communities and disseminate the information to interested audiences [14]. This model has been successfully used to educate physicians, nurses, and other health care service providers in the areas of Alzheimer's disease, STD/HIV prevention, alcohol abuse, and social services [14-17]. The TTT approach used alone or as part of a multifaceted campaign has demonstrated changes in knowledge, attitudes, and self-efficacy among health care providers [14,16,18].

We developed a CFS TTT curriculum to increase knowledge, self-efficacy, and management skills among workshop participants (e.g., core trainers) – primary care physicians, physician assistants, and nurse practitioners. The primary goal was for each core trainer to further reach 50 healthcare professionals in their field. The CFS TTT education program was developed in 2000 through an educational grant funded by CDC, administered through the Health Resources and Services Administration (HRSA) and issued to the CFIDS Association of America (CFIDS). Between 2000 and 2002, CFIDS worked with the Illinois Area of Health Education Centers (AHEC) Program to conduct the trainer training.

## Methods

We recruited primary care physicians, physician assistants, and nurse practitioners from HRSA AHEC sites across the United States. In 2001, invitations to participate in a two-day intensive workshop in Charlotte, North Carolina or Chicago, Illinois were sent to AHEC directors in six states: North Carolina, Texas, Florida, Oklahoma, Washington, and Utah. Consequently, to improve participation response, the Illinois AHEC director then sent letters to all 221 AHEC centers in the United States. In 2003, 113 invitations to participate in Dallas, Texas workshops were issued to previously interested individuals who asked to be added to a waiting list for future trainings. From this list we made the decision to screen participants for the Dallas workshop to better ensure that they could follow through with their commitment to train colleagues. We queried if they were physically able to conduct trainings; had local organizations (such as hospitals, medical or other health professional societies or universities) at which they could hold trainings; had contacts or relationships with these organizations; and were comfortable with public speaking and the use of A-V equipment.

The training program covered all participant costs (i.e., transportation, room and board) and participants received approximately 13 hours of CME upon successful completion. Participants in the 2001 training programs received a $250 stipend. Costs for individual workshops ranged from $10,000 to $25,000 and included travel expenses, instructor training and honoraria, personnel, materials, and facilities. The CFS TTT program represented a continuing medical education project, and similar to other TTT programs [14,15,19,20], ethics committee approval and informed consent was not sought as organizations partner with TTT programs to refer participants and participants gain continuing education credits.

The TTT program was developed by combining teaching methods and adult learning models to focus on three objectives: 1) knowledge; 2) self-efficacy; 3) and management skills, with the primary goal of conducing secondary presentations. The four training program goals are summarized in Table 1. All workshops followed a format that included an introduction session followed by didactic presentations, case study reviews, small group breakouts, presentation practice session, and a final question and answer session. Adult learning models constructs included using master trainers to teach the course (e.g., learning from a CFS professional expert), practicing diagnosing CFS in small groups, providing feedback on practice presentations, and recognizing the diverse backgrounds of the target audience in terms of subject matter.

**Table 1:** CFS Trainer-the-Trainer Workshop Goals

**Goal**	**Definition**
Knowledge	Define chronic fatigue syndrome
	Identify clinical diagnosis strategies
	Consider management approaches
	Recognize possible etiologies (contributing factors)

Self-Efficacy	Increase confidence in recognizing signs and symptoms of CFS
	Importance of using listening skills and non-judgmental responses

Skills	Collecting medical history, physical exam, and psycho-social data
	Make treatment and management recommendations based on symptoms

Secondary Presentations	Present to an audience of 50 colleagues and/or students trainers' home sites

The objective of the "CFS Diagnostic and Management Core" curriculum was to instruct core trainers as to evaluation, diagnosis, and management of CFS. Core trainers learned how to adapt and to utilize the educational model presented to them and following the workshop, they agreed to present one to two hour programs to groups of their peers. The evaluation employed quantitative and qualitative methods to capture outcome and process measures and focused on three program objectives: knowledge, self-efficacy levels, and secondary presentations.

Experts in CFS from the CDC, academic institutions, practitioners, and patient advocacy groups developed the course material. Specific workshop tools included: teaching script for a 30-minute didactic lecture; slides/overhead visuals; four individual case studies; decision-making models for diagnosis and treatment; a selected annotated bibliography; and copies of key journal articles. The educational program allotted for flexibility by allowing core trainers to vary case studies when making secondary presentations (i.e., 60-, 90-, or 120-minute presentations).

Following the workshop, core trainers were regularly informed of TTT program activities and new research findings through a monthly e-newsletter, which served as a reminder to plan and conduct educational sessions, as well as offer support and assistance for trainings. All participants had contact information for fellow participants and handouts and printed materials were provided free of charge for distribution at home sites.

The curriculum objectives were measured by the trainers' competency to perform the following: define CFS by the 1994 case definition [1]; recognize CFS symptoms and contributing factors; identify the wide-ranging impact of CFS on the patient, family, and society; and identify diagnostic and management strategies for CFS.

We used quantitative methods to measure the outcomes of knowledge and self-efficacy. Workshop participants completed an anonymous Course Evaluation and Assessment, the CFS Primary Care Provider Opinion Survey, the CFS Attitudes Test (CAT) [21], and 3) a CFS pre- and post- knowledge test. The Course Evaluation and Assessment was completed only as a post-test and had a total of 22 items, ranging from 1 to 5, with 1 being "poor" and 5 "excellent." Nine of these questions assessed self-efficacy expectation whereas the other questions were required for course evaluation (e.g., assessing course content and materials, quality of speakers, etc.). The CFS Primary Care Provider Opinion survey was specifically developed for use with the curriculum and includes 20 statements with 5 ratings (strongly agree; agree; undecided; disagree; and strongly disagree). The CAT survey is a 19-item instrument with a 7-point Likert scale (from strongly disagree -1 to strongly agree -7) that measures attitudes towards CFS; total scores range from 19 to 133 (higher scores reflect greater negative attitude towards CFS). The CAT includes 2 factors: a Responsibility factor (attitudes concerning the burden CFS, which is comprised of items 3, 5, 11, 18, and 19), and a Relevance factor (attitudes toward the validity of CFS, which is comprised of items 2, 4, 8, 12, and 16). The CAT has been shown to have moderate reliability and construct validity [19]. The CFS knowledge test, an objective test with a multiple choice format, was developed in part to meet accreditation requirements of the CDC CME accreditation office. Workshop participants had to score at least 70% on the post-test in order to receive continuing education credit. The knowledge test is linked directly to content in the curriculum. After the initial training session in Charlotte, the CFS knowledge test was increased in length and difficulty from 15 to 25 items for the Chicago workshop and decreased to 20 items for the Dallas workshop. SPSS was used to calculate descriptive statistics and paired t-tests for statistical significance.

Process evaluation data focused primarily on secondary presentations: the number of trainers trained, the number of secondary educational programs, the numbers of participants and target audiences in the secondary trainings, attendance at secondary trainings, course evaluations, and information on the most productive and active core trainers. In 2004, primarily qualitative mixed methods follow-up study was conducted with a subset of core trainers to provide additional data regarding secondary training sessions.

## Results

Seventy-nine core trainers completed the curriculum over a three-year period in five TTT workshops (see Table 2). (Due to missing data results are presented for 77 trainers.) Fifty-four practitioners (44 (81%) in response to AHEC recruitment letters and 10 (19%) recruited by word of mouth) attended the 2001 series of TTT sessions. For the 2003 workshops, we received replies from 63 persons for a response rate of 56%. Ultimately, a total of 23 people attended these two trainings with the remainder citing personal or scheduling conflicts, and a few individuals never responded. Core trainers participating in the workshops came from both rural and urban environments and represented diverse settings, including universities, professional societies, underserved communities, the military, and the government.

**Table 2:** Core Trainer Demographics (N = 77)*

**Characteristics**		**N**	**%**
Sex	Male	26	34
	Female	51	66

Age Range	21–30	5	6
	31–40	26	34
	41–50	31	40
	51–60	10	13
	61–70	5	6

Region	West	22	29
	Midwest	15	19
	South	27	35
	Northeast	14	18

Occupation	Physicians (MD, DO)	31	40
	Physician Assistants	11	14
	Nurse Practitioners/Nurses (PhD)	31	40
	Other^a^	4	6

Training Session	April 2001 (Charlotte, N.C.)	12	15
	May 2001 (Chicago, IL)	21	27
	August 2001 (Chicago, IL)	23	29
	July 2003 (Dallas, TX)	10	13
	September 2003 (Dallas, TX)	13	16

^a ^PharmD, PhD, Counselor, LSW, *missing data for 2 trainers

Two-thirds of the core trainers were women, and geographically, the majority of participants came from the South (35%) and the West (29%). Physicians and nurse practitioners (40% each) accounted for most of the trainers and only 20% were physician assistants. Six percent of core trainers suffered from and had been diagnosed with CFS, and 8% either had a family member with CFS or knew someone with the illness.

### Quantitative Results

#### Self-Efficacy and Knowledge Outcomes

All participants scored high on the knowledge level questions after completion of the two-day course and reported a high level of self-efficacy expectation in terms of the ability to recognize the signs and symptoms of CFS. Most core trainers showed high levels of self-efficacy expectation to the educational modules as measured by the Course Evaluation and Assessment post-test (Table 3). Eighty-two percent of core trainers responded "Very good" or "Excellent" when asked about their overall perceived self-efficacy in terms of diagnosing and managing CFS.

**Table 3:** Course Evaluation and Assessment Post-Test Survey (n = 73)

**Item**	**Mean**	**SD**
Confident able to define CFS	4.41	0.72
Confident discuss contributing factors to CFS	4.25	0.76
Confident can categorize CFS as an illness	4.44	0.69
Confident identify myths around CFS	4.71	0.55
Confident describe diagnostic process	4.40	0.74
Confident identify management strategies	4.49	0.65
Confident recognize unpredictability of symptoms	4.33	0.63
Confident recognize impact of CFS	4.55	0.73
Confident discuss disability issues of CFS	4.50	0.72
Total self-efficacy score	4.40	0.63

Data from the Chicago workshops showed an improvement from pre-test to post-test (p < 0.01) on the total Primary Care Opinion Survey. There were significant differences for the following items: "Confident in ability to diagnosis CFS" (p < 0.001); "CFS manifests more through psychological symptoms than through physical symptoms" (p < 0.01); "I do not doubt that CFS is a distinct syndrome" (p < 0.05); "I suspect CFS is just another form of depression" (p < 0.05); and "I feel that continuing education on CFS will influence my clinical practice" (p < 0.05).

Significant differences were found for the CAT Responsibility and Relevance factors in the May 2001 Chicago workshop (see Table 4). At the post-test core trainers had less negative attitudes towards persons with CFS and viewed CFS in a more positive manner. While not significant, the mean score for the total CAT decreased from 42.0 to 38.0 (p = 0.13), which shows an improvement in attitudes towards CFS. For the Responsibility factor, the mean sum for trainers decreased (i.e., improved) from 8.6 to 6.7 (p = 0.03) on the post-test. The Relevance factor mean sum decreased (i.e., improved) from 13.3 on the pretest to 10.7 on the post-test (p = 0.04). A significant difference on the total CAT score was seen for both Chicago workshops (n = 44) combined, p < 0.01, as well as for the Responsibility (p < 0.05) and Relevance (p < 0.05) factor (data not shown).

**Table 4:** Chicago May 2001 CAT Survey Pre- and Post-test Results (n = 21)

	**Pre-test** **Mean (SD)**	**Post-test** **Mean (SD)**	**p-value**
Overall CAT score	42.0 (11.7)	38 (10.9)	0.13

Responsibility Factor	8.6 (4.12)	6.7 (2.1)	0.03

Relevance Factor	13.3 (5.4)	10.0 (4.5)	0.04

Results from the test of CFS knowledge survey in both the Chicago (n = 44) and Dallas (n = 21) workshops show that trainers demonstrated a significant (p < .001) improved performance from the pre-test to the post-test. In Chicago, for example, on average, participant knowledge of CFS increased by 5 items or approximately 20%. On the post-test, 41 (93%) of the 44 trainers correctly answered 20 (80%) of more of the 25 knowledge questions.

In the Dallas workshops, 6 (29%) participants answered 80% of 20 knowledge questions correctly at pre-test compared to 16 (76%) at post-test. Core trainers from the Dallas workshops showed significant improvement in CFS knowledge from the pre- to post-test knowledge survey (see Table 5). The pre-test mean 14.5 (SD = 2.1) increased to 16.8 (1.7) on the post-test (p = 0.001).

**Table 5:** Dallas 2003 Workshops CFS Knowledge Survey Pre- and Post-test Results (n = 21)

	**Pre-test** **Mean (SD)**	**Post-test** **Mean (SD)**	**p-value**
CFS Knowledge score	14.5 (2.2)	16.8 (1.7)	0.001

### Qualitative Results

#### Secondary Presentations

Following the trainer workshops, between April 2001 and October 2005, 28 of the 79 core trainers conducted a total of 50 peer education sessions reaching 2,064 participants. Forty-nine trainers never conducted an educational program. Audience size ranged from 6 to 250 people. Three trainers were responsible for 20 (40%) of the 50 post-workshop educational sessions. The audiences to which these trainers presented educational forums consisted mainly of physician assistants and students, nurses and nurse practitioners, and occupational therapists.

In 2004, valid contact information for 58 core trainers allowed for a follow-up survey. Nineteen of those contacted agreed to complete a quantitative and qualitative survey for a 33% response rate. This population included 6 physicians (33%), 6 nurses (33%), 5 physician assistants (27%), and one PhD (5%). At least nine of the respondents held academic positions and one professional had a diagnosis of CFS. Of the nineteen respondents, 12 (66%) were female and 6 (33%) male.

Overall the workshop material was well received and deemed to be at the appropriate education level. The respondents also stated that generally the secondary educational sessions were a positive experience. Qualitative process data collected from these individuals was categorized into the following themes: audience, barriers to conducting trainings, and future trainings.

#### Audience

Many core trainers reported positive experiences in conducting the secondary CFS educational modules. Audiences responded with "excellent comments," "excellent response and questions," and "healthcare workers were very receptive to the information provided." However, physician assistants, nurses, social workers and students were perceived by core trainers as more interested than physicians in learning about CFS. Core trainers stated that this group appeared to be engaged, ask questions, and were receptive to the information presented.

"It was favorable and well received by my PA students."

"I am most confident providing the CFS curriculum to a PA student audience."

"I usually have large audiences of RN's from 20 to 100. The group is very interested in the topic and tell me after the presentation that they had no idea what CFS was like."

"Nurses were very receptive."

"I have been fortunate to have PA students and PAs as most of my audiences. They are open to the information and have excellent questions."

In terms of physicians, several trainers expressed that physicians were less likely to treat CFS as a real illness, and one trainer commented:

"When I have trained a mixed audience that included physicians, the experience was less positive with physicians having more of an attitude that CFS is not real. I needed to remind them what was thought of H. pylori when that theory was first presented."

#### Barriers to conducting trainings

Two barriers emerged regarding the trainers' ability to conduct educational modules. First, core trainers cited difficulties securing opportunities to conduct CFS trainings. For example, workplace policies impeded the organization and administration of CFS trainings. Some core trainers commented that while the TTT sessions provided excellent information about the diagnosis and management of CFS, participants did not receive adequate guidance on how to incorporate or initiate the curriculum into their university or hospital environment. In some cases trainers cited grand rounds opportunities as directly related to their ability to penetrate into an educational forum at a university or hospital. However, these same participants expressed the need for guidance in obtaining proper administrative authorization, information to advocate for CFS on hospital education committees, and additional training on modifying the CFS curriculum for grand rounds presenters. For instance, one trainer cited logistical limitations in clinical practice schedules as a potential barrier to arranging an educational session. Others commented specifically on Grand Rounds.

"I offered to do a Grand Rounds but my hospital education committee did not respond."

"I am awaiting clearance to deliver a Grand Rounds – currently there are very few people authorized to deliver Grand Rounds."

"[Need to] indicate what additional criteria need to be satisfied to permit course presentations to become providers of Grand Rounds."

A second barrier to conducting trainings for some participants was the lack of experience with CFS and therefore lack of confidence in holding a CFS educational session. For example, participants expressed being uncomfortable with explaining the etiology or cause of CFS; CFS management strategies; and evaluation of treatment strategies as barriers for not being able to conduct a session.

"I think you need to train people with some [CFS] experience or medical background to be able to talk knowledgeably with an audience and answer questions. The trainer books were good but I felt I was talking about something I really did not know about."

"More focus on the etiology and evaluation of management approaches."

"More information to prepare presenters to answer difficult questions that are beyond our train-the-trainer skills and clinical/research skills."

"I do not feel that I can comfortably field CFS management questions from a seasoned clinician audience."

"The area I feel the least comfort is in describing the etiology of cause of this illness syndrome and describing emerging management strategies and to evaluate their efficacy."

"I have not felt comfortable with my level of experience with CFS to talk confidently."

#### Future trainings

The core trainers provided valuable feedback based on their original training session and experiences from holding educational sessions in the field. In terms of core training, it was suggested to include an educational component on how to address difficult or negative questions from audience members. One suggestion was to include a frequently asked difficult question list. A second suggestion was to modify and tailor some of the curriculum modules depending on the audience. For example, delivery of information for physician assistants and nurse practitioners may differ from information provided to physician audiences.

When asked about the content material from the core training, some participants replied that additional information in the following areas would be helpful for presentations: physiologic definition of CFS; etiology of CFS in relation to tick-borne infections, mold, and toxin exposure; and management strategies and evaluation methods for efficacy of treatments. Finally, core trainers mentioned the need to stay informed about current research. Suggestions included posting information on a third-party professional education website, sending email updates, or holding follow-up seminars.

## Discussion

The CFS TTT program demonstrated success in meeting the program goals of increasing knowledge about CFS and raising core trainers' self-efficacy expectation in recognizing signs and symptoms of the illness. While not all the core trainers met the objective of reaching 50 individuals, as a group they successfully reached 2064 primary care providers. Trainers showed significant differences from pre-test to post-test for the Primary Care Provider Opinion Survey, the CAT, and the CFS Knowledge test demonstrating that provider attitudes and knowledge towards CFS diagnosis and management improved. Specifically, negative attitudes towards CFS improved (p < .01) on the CAT and for each of the Responsibility and Relevance factors.

Core trainers scored high on post-training knowledge and self-efficacy expectation tests with scores for knowledge measures generally higher than self-efficacy. For example, the highest means were 4.71 for *identifying myths*, 4.55 for *recognizing impact of CFS*, and 4.50 *discuss disability issues of CFS*, whereas lower mean scores were identified for self-efficacy expectation measures (i.e., diagnostic process of identifying CFS): *discuss contributing factors to CFS *(4.25), *recognize unpredictability of symptoms *(4.33), and *describe diagnostic process *(4.40). Additionally, analyses from the Chicago and Dallas workshops demonstrated significant changes for program participants in CFS knowledge.

Program participants were able to increase knowledge about CFS and responded that they would be able to recognize new cases, yet it appears that applying the knowledge gained to diagnosing and managing CFS remains a challenge. Self-efficacy expectation scores from a follow-up survey with a subset of core trainers further support this finding. When asked about being prepared to deliver secondary presentations, these participants had a mean score of 3.63. Qualitative data from some trainers provide plausible explanations as to why self-efficacy dropped after the training: some core trainers were not comfortable presenting secondary educational sessions because they could not describe the etiology or cause of CFS, whereas others had limited knowledge of management strategies, or did not possess a high level of experience with CFS patients.

The impact of the TTT program in terms of knowledge gains was consistent with outcomes from other TTT programs [14-16] and demonstrates that a CFS provider education is feasible despite complexities associated with CFS. CFS is difficult to diagnosis and treat, and there are no biomarkers for CFS making detection difficult. The lack of a diagnostic test and evolving criteria for diagnosis and management add to the pre-existing skepticism among providers. The ultimate goal of decreasing morbidity through increased detection and better management therein lies with changing provider behavior, specifically, the ability of providers to recognize and diagnosis CFS.

Another goal for core trainers was to return to their home sites and reach 50 individuals by conducting secondary educational sessions. While only one-third of the core trainers accomplished this objective, over a two-year period a total of 2064 individuals ultimately received training and this achievement is comparable to the TTT secondary training of 3276 individuals in a dementia program [14] and 2066 trainees reached in an alcohol abuse program [16].

Process evaluation data yielded important information regarding secondary course implementation and suggests two barriers to the secondary CFS education. First, physicians were not terribly receptive to secondary training. Only 7 of the 28 core trainers who conducted educational sessions were physicians and only 6 of the 50 secondary trainings had audiences comprised solely of physicians. Although the core trainer physicians increased personal knowledge and self-efficacy skills in the training workshop they found it difficult to further disseminate information to their colleagues. In an evaluation of a physician TTT program, VanGeest similarly found that while physician trainers possessed content knowledge they found it difficult to teach skills to colleagues [22]. Anecdotal evidence suggests that physicians may be more accustomed to traditional education methods received in their medical school and residency training programs. These include, for example, courses with simulated patients in the third and fourth years of medical school, Grand Rounds presentations, and case studies in clinical rotations. Therefore, CFS education of physicians may be best accomplished by reaching physicians through medical school curriculums and residency training programs. Additionally, given the advent of technology, offering CME courses through the Internet or adding CFS diagnostic algorithms to handheld PDA's, enables physicians to further educate themselves without sacrificing valuable time.

Alternatively, the format was more acceptable for physician assistants and nurse practitioners or nurses. Three trainers with such backgrounds educated 50% (1016) of all individuals. Furthermore, most (88%) of the 2064 people who received secondary CFS training from a core trainer were physician assistants, nurse practitioners, students, or educators. Qualitative feedback further elucidate this finding with a report of a "less positive" experience in the CFS education program when physicians were part of the audience, and that physician assistants, nurse practitioners, and students were more interactive during the presentations than their physician counterparts.

Second, workplace policies and obtaining administration authorization for trainings proved to be a barrier for some participants. Although the TTT education workshop did not provide in-depth information on health education policies or access to hospital education committees, assistance with organizing secondary education programs was repeatedly offered through AHEC and CFIDS. Some participants lacked knowledge in how to petition their institutions' educational committee or approach departments to organize grand round opportunities. Others cited time constraints or conflicts in the administrative aspects of procuring secondary presentation opportunities. Process evaluation data show that for core trainers able to conduct secondary educational sessions, many used connections or built-in audiences such as universities, student audiences, or professional society affiliations.

This research has several limitations. The challenges of evaluating this type of training for CFS at the time it was implemented must be considered. Only one survey instrument of moderate reliability was available to assess CFS specific attitudes. Other instruments to specifically measure CFS knowledge (i.e., success in mastering the core curriculum) were non-existent and thus developed through the continuing education accreditation process. However, other TTT studies have used similar illness appropriate knowledge measures to evaluate programs [14-17]. Measurement of knowledge and secondary presentations was less of a challenge than self-efficacy. The measurement of knowledge outcomes was feasible though survey instruments, whereas the measurement of self-efficacy expectation may have been premature given the continual evolution of CFS clinical guidelines, i.e., the evolving diagnostic algorithm. Rather, the use of mixed evaluation methods that include quantitative (i.e., pre- and/or post-test surveys, process evaluation data) and qualitative data present more valuable information and insight for evaluation of the CFS TTT workshops. Additionally, strategies for optimal program implementation involved improving outcomes measures from one workshop to the next.

The core trainers who volunteered to participate in the workshop training do not represent a random sample. The sample selection of core trainers has two limitations as participants were selected in part because of: 1) CFS interest, and 2) ability or opportunity to secure secondary teaching opportunities. Eleven of the 79 core trainers either had received a diagnosis of CFS (n = 5) or knew some who had CFS (n = 6), and may have participated for personal reasons and may have been more highly motivated to complete the workshop.

A second limitation was the selection of volunteers who were affiliated with teaching institutions or had teaching experience. For the first series of workshops, AHEC sent letters to member centers soliciting volunteers interested in learning about CFS. Many AHEC centers are affiliated with academic institutions and provide ample opportunities for secondary instruction. Recruitment for the second series of workshops in part selected individuals based on ability to conduct educational programs. For example, the three core trainers responsible for 40% of the "home site" trainings all were educational instructors at universities. Thus, the core trainers may not represent the audiences they would further educate.

The qualitative data was obtained from a non-random sample of the original trainers. Due to missing information, contact information was available for 58 of the 79 core trainers. Of those with valid contact information, many were busy working professionals with limited time. However, the sample is reflective of the original 79 core trainers as measured by the equal representation of sex and occupation. Two of the respondents gave permission to complete only to the qualitative portion of the survey based on their disagreement with the course content. In particular, each felt the TTT session should have included more information on alternative hypotheses for CFS etiology.

Results from this project have informed Phase II of the CFS provider education project. CFS education for physicians now includes grand rounds presentations developed using evidence-based research. A few of the original trainers still conduct secondary educational sessions reaching physician assistants, nurse practitioners, and allied health professionals. Additionally, materials from the original workshop have been revised and offered as a self-study continuing education course. Medical professionals can now receive free continuing education credits through the CDC by completing an on-line CFS continuing education program.

## Conclusion

Results from the CFS TTT program inform CFS education of primary care providers in four ways. First, the CFS TTT program illustrates the merits of a collaborative partnership between government organizations (e.g., the CDC and AHEC) and a patient advocacy group (CFIDS) in the ability to develop an educational program aimed at primary care providers. AHEC through a joint collaboration with CFIDS recruited 79 health care professionals to become core trainers and participate in a CFS workshop. Representation of the core trainers was diverse in terms of occupation, geographic region, age, and gender.

Second, the program was successful in increasing CFS knowledge and perceived self-efficacy skills among physicians, physician assistants, and nurse practitioners. However, while post-test scores for self-efficacy expectation were high, qualitative data suggest that over time maintaining this skill was a challenge. Future TTT programs may wish to address how to maintain knowledge and skills learned in the program by offering a periodic booster course or encouraging program participants to communicate with one another to increase confidence. Further research regarding an on-line education review or certification to boost behaviors is also warranted.

Third, valuable information about the secondary educational sessions conducted by trainers was gained through the follow-up study. Although some primary care providers participated in the TTT program and did well, not all core trainer audiences may be receptive to the TTT method. While the CFS TTT program demonstrated that diverse populations of primary care providers can participate together in an educational program and benefit from the curriculum, it also showed that secondary target audiences may respond differently to the type of education instruction. Programs developing TTT may wish to pursue additional needs assessment on the intended secondary target audiences before training and educating core trainers, and conduct process evaluations to gain insight into the program implementation.

Finally, administrative constraints may impede completion of secondary presentations. Core trainers often faced administrative barriers in their efforts to conduct secondary educational sessions and future TTT programs are encouraged to examine the process of implementing secondary trainings before offering the TTT session. For example, if feasible, one solution would be to assist directly in the organization and scheduling of secondary trainings. Alternatively, a section of the TTT curriculum could focus on how core trainers contact and arrange for Grand Rounds lectures.

## Competing interests

The authors declare that they have no competing interests.

## Authors' contributions

DB drafted the manuscript and participated in the statistical analysis. KM, TL, and KF participated in the study design and coordination and helped to draft the analysis. KH performed the statistical analysis and participated in study coordination and design. WR conceived of the study, participated in study coordination and helped to draft the manuscript. All authors read and approved the final manuscript.

## Pre-publication history

The pre-publication history for this paper can be accessed here:


